# The Effects of Lentinan on the Hematological and Immune Indices of Dairy Cows

**DOI:** 10.3390/ani14091314

**Published:** 2024-04-27

**Authors:** Lun Tan, Caiyun Fan, Dian Wang, Xiao Li, Meng Wang, Zhao Zhuo, Shuaihong Li, Yuhang Ding, Zixi Yang, Jianbo Cheng

**Affiliations:** 1 College of Animal Science and Technology, Anhui Agricultural University, Hefei 230036, China; tanlun0713@163.com (L.T.); fancaiyunnmgbt@163.com (C.F.); 13685693231@163.com (X.L.); wm13685659189@163.com (M.W.); zhuozhao90@163.com (Z.Z.); shuaishuaipianer@163.com (S.L.); dingyh01041113@163.com (Y.D.); yzxnanjing@163.com (Z.Y.); 2 Inner Mongolia Youran Dairy Group Limited, Hohhot 010010, China; wd@yourandairy.com; 3 National Center of Technology Innovation for Dairy, Hohhot 010010, China

**Keywords:** lentinan, anti-inflammatory, dairy cows, metabolome

## Abstract

**Simple Summary:**

Lentinan is a polysaccharide extracted from mushrooms, which could have anti-inflammatory and antioxidant properties. It is widely used in human medicine while rarely in husbandry. Previous studies have shown that early-lactation cows suffer from a negative energy balance, which could lead to various inflammations. In this study, we aimed to investigate the effects of lentinan on immune ability in dairy cows. Some factors that are prone to causing inflammation in cows, such as IL-2, IL-8, and some liver injury markers, such as AST and ALT, were reduced after adding lentinan. In addition, metabolites revealed that lentinan upregulated some immune-beneficial metabolites in plasma, including some quinones and trehalose, which can promote anti-inflammatory and antioxidant effects. The findings provide a basis for the use of lentinan as a new type of cow additive.

**Abstract:**

In this study, we investigated the effects of lentinan (LNT) on hematological parameters, immune indices, and metabolite levels in dairy cows. We randomly assigned forty Holstein cows to four treatment groups. The treatments consisted of 0, 5, 10, and 15 g/d of LNT. Compared with the control group, the addition of 10 g/d of LNT decreased the content of ALT and IL-8 but simultaneously increased the content of IL-4 in the cows’ serum. Supplementation with 10 g/d of LNT decreased the levels of lymphocyte, RDW, ALT, AST, TC, IL-2, and IL-8, but, concurrently, in-creased the levels of granulocytes and IL-4 in their serum. In addition, supplementation with 15 g/d of LNT decreased the levels of RDW, TC, IL-2, and IL-8, but, at the same time, increased the levels of IL-4 and IgM in their serum. For the metabolomic analysis, cows fed with 0 and 10 g/d of LNT were selected. The results showed that 10 metabolites, including reduced nicotinamide riboside and trehalose, were upregulated in the 10 g/d group. These differential metabolites were enriched in tyrosine metabolism and trehalose degradation and altered two metabolic pathways of ubiquinone and other terpene quinone biosynthesis, as well as starch and sucrose metabolism. These findings provide evidence that LNT could be used to reduce the risk of inflammation in dairy cows.

## 1. Introduction

Lentinan (LNT) is a type of β-1, 3-glucan with β-1, with six branches, that is obtained from *Lentinus edodes*. It is considered a new functional food and nutritional health care product due to its low toxicity and immune-regulatory and anti-tumor characteristics [1,2]. As the main component of lentinan, β-glucans play a key role in immune regulation, and the mechanism of how β-glucans activate the immune system is complex and has not yet been fully elucidated [3]. In general, they can trigger downstream signals by binding to pattern recognition receptors, complement receptors, and other membrane receptors, thereby activating the functions of immune cells, especially Dectin-1, which is the most important β-glucan receptor as it can be highly expressed in macrophages and neutrophils [3,4]. Overall, β-glucans can enhance the specific and non-specific immunity of the human body. Currently, there is a large number of studies related to the immunomodulatory effect of β-glucans [5]. Jo et al. found that one β-glucan, extracted from Cordyceps militaris fruiting bodies, could directly activate leukocytes and further stimulate their phagocytosis, cytotoxicity, and antibacterial activity in vitro [6]. Some in vitro studies have shown that β-glucans can directly activate leukocytes and further stimulate their phagocytosis, cytotoxicity, and antibacterial activity [6]. In addition, β-glucans have been demonstrated to inhibit the secretion of proinflammatory mediators such as TNF-α, IL-1α, and NO, which are considered as important chemical messengers that induce numerous biological reactions [7]. In addition, Ahn et al. reported that LNT could attenuate IL-1β and IL-18 secretion and reduced endotoxin lethality via the inhibition of non-canonical inflammasome activation in an inflammatory mouse model [8]. Similarly, Ren et al. reported that LNT preparations were able to reduce serum TNF-α and IL-1β and pancreatic hydroxyproline in an inflammatory mouse model, effectively inhibiting the progression of inflammation [9]. In another study, LNT was found to possibly inhibit the degradation of IκBα and downregulate the expression levels of TNF-α, IL-1β, IL-6, and IL-8 in fish cells, indicating that LNT can inhibit the NF-κB signaling pathway to produce anti-inflammatory effects [10].

In ruminants, β-glucans can reduce the level of white blood cells in milk to reduce the risk of mastitis, increasing the concentration of immunoglobulins in serum to enhance the adaptive immune system of Holstein calves [11]. However, the utilization of β-glucans, especially LNT, is still far too scarce. Dairy cows are prone to inflammation during the peak lactation period, resulting in negative impacts on their production performance [12]. This study aims to explore whether supplementation with LNT affects the hematological parameters and cytokine levels in the blood of high-yielding dairy cows, combined with a metabolomic analysis, to explore the effect of LNT on improving the immunity of high-yield dairy cows.

## 2. Materials and Methods

The experimental protocol and sample collection were carried out in accordance with the Regulations on the Administration of Laboratory Animals implemented by the National Science and Technology Commission of the People’s Republic of China. The experiment was approved by the Committee of the College of Animal Science and Technology, Anhui Agricultural University, Hefei, China (No. SYDW-P20190600601).

### 2.1. Animals and Experimental Design

The experiment was conducted at Nanqiao Dairy Farm in Chuzhou, China. Forty lactating Holstein cows with similar initial days in milk (57 ± 21.3), milk yield (42.3 ± 7.88), and parity (3.44 ± 1.45) were randomly divided into four groups, with ten cows in each group. The control group (CON) was fed a basal diet without LNT addition, and the three other treatment groups were fed a basal diet with 5 g/d (LL: low-dose LNT), 10 g/d (ML: middle-dose LNT), and 15 g/d (HL: high-dose LNT), respectively (dry matter basis). The cows were housed in tie stalls, had free access to fresh water, and received total mixed rations (TMR; concentrate-to-forage ratio of 40.9:59.1; based on NRC 2001) twice a day at 09:00 and 15:00 [13]. The TMR ingredients and nutrient components are listed in Table 1. The lentinan (>35.99% purity) was provided by Anhui Fengcao Biotechnology Co., Ltd. (Hefei, China). The animal trial lasted for eight weeks.

### 2.2. Sample Collection

Blood and feed samples were collected on the 56th day of the trial. Before morning feeding, 10 mL of blood from the samples was placed into EDTA-treated tubes and analyzed to assess the hematological parameters. Next, 10 mL of blood from the sample was added to vacutainer tubes without anticoagulants and 10 mL of blood from the samples was place into heparin sodium-treated tubes. Then, the blood samples were then centrifuged (TD5, Shanghai Lu Xiangyi Centrifuge Instrument Co., Ltd., Shanghai, China) at 3000× *g* for 15 min at 4 °C to obtain serum and plasma, and stored at −80 °C for analysis of the blood biochemical indicators, immune factors, antioxidant indicators, and plasma metabolome.

### 2.3. Hematological Parameter, Blood Biochemical Indicator, Serum Immunologic Factor, and Antioxidant Indicator Analyses

The hematological parameters, including white blood cell count (WBC; reference value 5.0–16.0 × 10^9^/L), monocyte (Mon; reference value 0.3–1.6 × 10^9^/L), lymphocytes (Lymph; reference value 1.5–9.0 × 10^9^/L), granulocytes (Gran; reference value 2.3–9.1 × 10^9^/L), red blood cell count (RBC; reference value 5.00–10.10 × 10^12^/L), hemoglobin (HGB; reference value 90–139 g/L), hematocrit (HCT; reference value 28.0–46.0%), mean red blood cell volume (MCV; reference value 38.0–53.0 fL), mean corpuscular hemoglobin (MCH; reference value 13.0–19.0 pg), mean corpuscular hemoglobin concentration (MCHC; reference value 300–370 g/L), red blood cell distribution width (RDW; reference value 14.0–19.0%), platelets (PLT; reference value 120–820 × 10^9^/L), mean platelet volume (MPV; reference value 3.8–7.0 fL), platelet distribution width (PDW; reference value 14.0–19.0), and platelet hematocrit (PCT; reference value 0.12–0.42%) were analyzed using a SYS-MEX XT-4000i Automated Hematology Analyzer (Sysmex, Kobe, Japan). The reference values of the indices above are according to the Merck Veterinary Manual (2016) and Veterinary Internal Medicine (1999) [14,15].

Alanine aminotransferase (ALT), aspartate transaminase (AST; reference value 50.0–150.0 U/L), alkaline phosphatase (ALP; reference value 28.0–233.0 U/L), total protein (TP; reference value 62.0–80.0 g/L), albumin (ALB; reference value 25.0–35.0 g/L), globulin (GLB; reference value 30.0–49.0 g/L), urea (reference value 3.60–9.30 mmol/L), glucose (GLU; reference value 3.11–4.89 mmol/L), calcium (Ca; reference value 2.00–3.00 mmol/L), phosphorus (P; reference value 1.30–2.80 mmol/L), total cholesterol (TC; reference value 1.20–5.20 mmol/L), and total glyceride (TG; reference value ≤ 0.10 mmol/L) in the cows’ serum were determined using an automatic biochemistry analyzer (Synchron CX5 Pro, Beckman Coulter, Fullerton, CA, USA). The reference values of the indices above are according to the Merck Veterinary Manual (2016) and Veterinary Internal Medicine (1999) [14,15].

The determination of seruminterleukin-1β (IL-1β), interleukin-2 (IL-2), interleu-kin-4 (IL-4), interleukin-6 (IL-6), interleukin-8 (IL-8), interleukin-10 (IL-10), interleu-kin-11 (IL-11), immunoglobulin A (IgA), immunoglobulin G (IgG), immunoglobulin M (IgM), transforming growth factor-β1 (TGF-β1), tumor necrosis factor-α (TNF-α), superoxide dismutase (SOD), glutathione peroxidase (GSH-Px), and malonaldehyde (MDA) was carried out using the corresponding bovine enzyme-linked immunosorbent assay (Elisa) kit (Shanghai Enzyme-linked Biotechnology Co., Ltd., Shanghai, China).

### 2.4. Plasma Metabolomic Analysis

We added 400 µL methanol to 100 µL plasma and vortex mix (Vortex-Genie2, Scientific Industries, New York, NY, USA), centrifuged the mix at 4 degrees and 12,000 rpm for 10 min, transferred the supernatant into a 1.5 mL centrifuge tube, and evaporated it with a centrifugal concentration instrument (Genevac miVac, Tegent Scientific Ltd., Ipswich, UK). Lastly, it was dissolved with 100 µL of 1% acetonitrile and the supernatant was removed for testing (LC-MS).

MarkerView 1.3 (AB Science, Concord, ON, Canada) software was used to extract the peak area, mass charge ratio, and retention time of primary mass spectrometry raw data to generate a two-dimensional data matrix. PeakView 2.2 (AB Science, Concord, ON, Canada) was used to extract the secondary mass spectrometry data and compare them with the Metabolites database, the HMDB, METLIN, and standard to identify the metabolite ID and assign the obtained ID to the corresponding ions in the two-dimensional data matrix of primary mass spectrometry. Statistical and path analyses of the identified data were performed using a self-compiled program based on R language.

### 2.5. Statistical Analysis

Principal component analysis (PCA) and orthogonal partial least-squares discriminant analysis (OPLS-DA) were used to reflect inter-group differences. During this analysis, the VIP value of all metabolites was obtained. In general, a VIP > 1 is a necessary condition for a metabolite to be considered as a biomarker. Based on pathway-associated metabolite sets (SMPDB) (October 2019), the metabolite set enrichment analysis of differential metabolites was conducted, and the test method used was a hypergeometric test in over-representation analysis (ORA). Based on the KEGG, metabolic pathway analysis of differential metabolites was conducted by using the hypergeometric test (ORA) and relative betweenness centrality.

We selected differential metabolites and took the differential hematological parameters, blood biochemical indicators, and immunologic factors as environmental factors. We then calculated the Spearman rank to reflect their correlation. The obtained numerical matrix was visually displayed through a heatmap diagram, and the matrix with a correlation |r| value > 0.5 and a *p*-value < 0.05 was marked with an asterisk, *, and * represents 0.01 < *p* ≤ 0.05, ** represents 0.001 < *p* ≤ 0.01, and *** represents *p* ≤ 0.001.

All data statistics were performed using SPSS Statistics (version 22, IBM, New York, NY, USA). The hematological parameters, blood biochemical indicators, serum immunologic factors, and antioxidant indicators were analyzed through one-way ANOVA, with statistical significance defined as *p* < 0.05 and a tendency to be defined as 0.05 < *p* < 0.10.

## 3. Results

### 3.1. Effect of LNT Supplementation on the Hematological Parameters of the Dairy Cows

As shown in Table 2, compared with the CON group, the proportion of lymphocytes decreased (*p* = 0.023), while the percentage of granulocytes increased significantly in the ML group (*p* = 0.018). RDW decreased significantly in the ML and HL groups (*p* = 0.026). In addition, supplementation with LNT showed a tendency to decrease the proportion of lymphocytes (*p* = 0.069).

### 3.2. Effect of LNT Supplementation on the Serum Biochemical Indices of the Dairy Cows

The effect of LNT supplementation on the serum biochemical indicators of the dairy cows is shown in Table 3. Compared with the CON group, the level of ALT decreased in the LL and ML groups (*p* < 0.001). AST decreased in the ML group (*p* = 0.020) and TC decreased in the ML and HL groups (*p* = 0.014). In addition, in the LL group, the TP content increased (*p* = 0.029) and globulin showed an increasing trend (*p* = 0.074).

### 3.3. Effect of LNT Supplementation on the Serum Immune Indices of the Dairy Cows

The serum immune indices of the dairy cows are listed in Table 4. Compared with the CON group, the level of IL-2 decreased in the ML and HL groups (*p* = 0.027). In contrast, IL-4 increased but IL-8 declined in the LL, ML, and HL groups (*p* = 0.039; *p* = 0.026). In the HL group, the level of IgM decreased (*p* = 0.005). Furthermore, supplementation with LNT showed a tendency to decrease the level of IL-1β, IL-6, and TNF-α (*p* = 0.070; *p* = 0.099; *p* = 0.077), and it also showed a tendency to increase the level of IL-10 (*p* = 0.083).

### 3.4. Effect of LNT Supplementation on Serum Redox Indices of the Dairy Cows

As shown in Table 5, supplementation with LNT had no significant effect on the content of SOD, GPX, and MDA in the serum of the dairy cows (*p* > 0.1).

### 3.5. Metabolomic Analysis of Blood Samples

**Figure 1:**
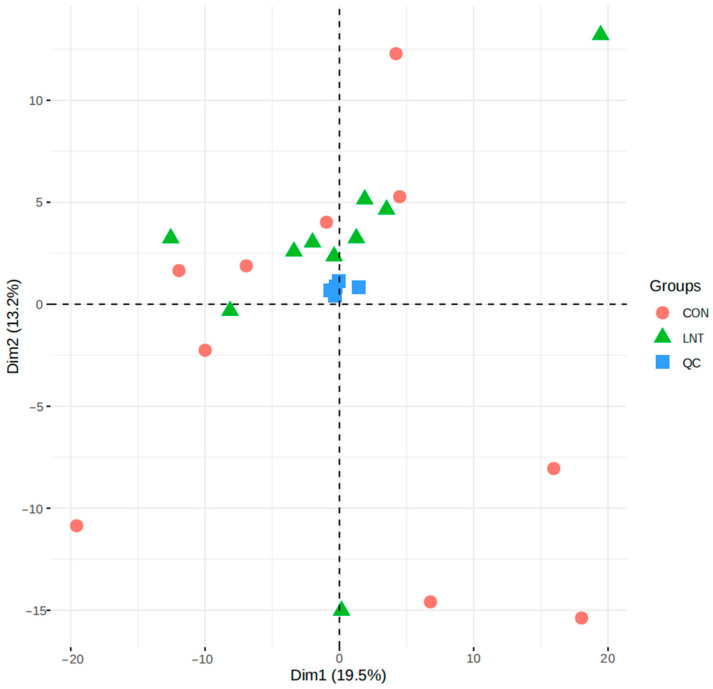
Principal component analysis diagram of the quality control samples.

**Figure 2:**
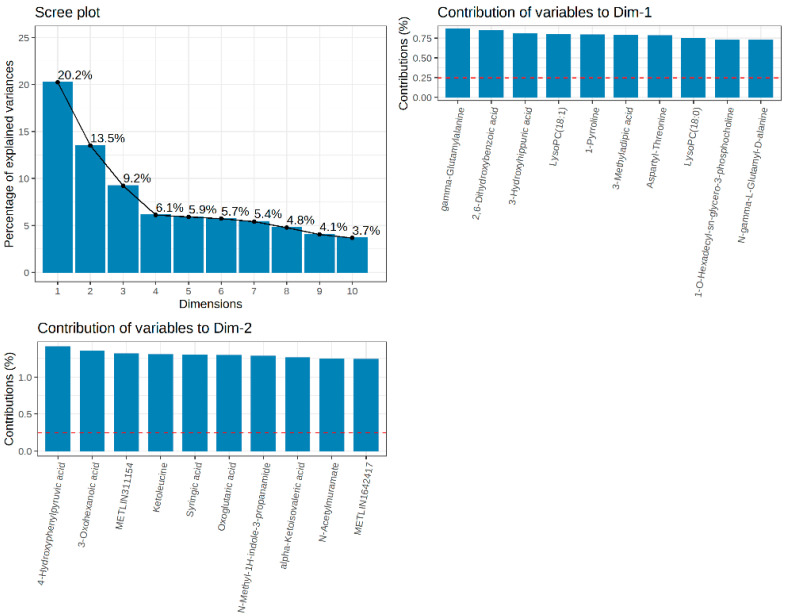
The scree plot in the upper left corner shows the proportion of the variance in the main component decomposition to the X variable. The loading plot shows the top 10 variables with the highest load on each principal component.

A total of 67 types of metabolites were identified from 20 plasma samples, of which amino acids were identified in the highest number of types (98). The principal component analysis (PCA) chart of the quality control samples (Figure 1) shows that, in the combined QC samples, which showed stable signals and high data quality during the testing process, there was no significant outlier between the two groups of samples and there were specific differences in the first two principal dimensions. As shown in Figure 2, dimension 1 and dimension 2 account for 33.8% of the explained variance, and the top metabolite with the greatest contribution to dimension 1 was gamma-glutamylalanine, and the top metabolite with the greatest contribution to dimension 2 was 4-hydroxyphenylpyruvic acid.

**Figure 3:**
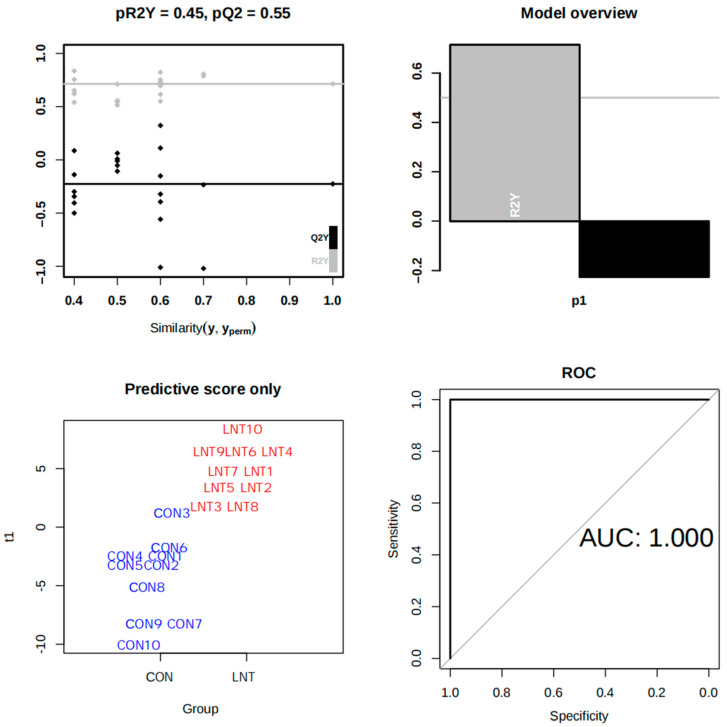
Orthogonal partial least-squares discriminant analysis (OPLS-DA) plot. Sensitivity: 0.90; specificity: 1.00; negative level: CON; positive level: LNT.

We further visualized the differences in metabolites between the different groups through an orthogonal partial least-squares discriminant analysis (OPLS-DA). The results are shown in Figure 3, and the distinction between the LNT group and the CON group was satisfactory, with the AUC = 1.00. Of note, the model shows signs of overfitting (pR2Y = 0.45; pQ2 = 0.55).

### 3.6. Differential Metabolites between the LNT and CON Groups

**Figure 4:**
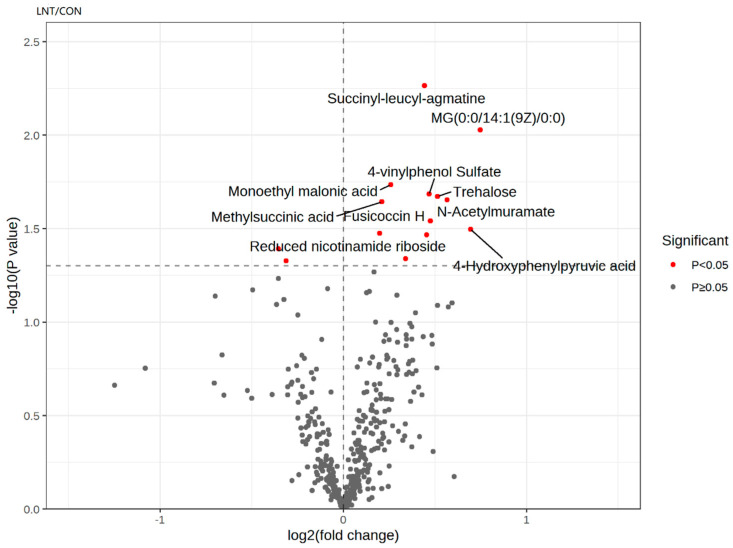
Volcano plot showing the statistical significance (*p*-value) and change range (fold change) of metabolites. Only the 20 metabolites that meet the *p*-value and multiple change conditions are marked most significantly.

As shown in Figure 4, reduced nicotinamide riboside, 4-hydroxyphenylpyruvic acid, 4-vinylphenol sulfate, fusicoccin H, methylsuccinic acid, MG(0:0/14:1(9Z)/0:0), monoethyl malonic acid, N-acetylmuramate, succinyl-leucyl-agmatine, and trehalose represent the significantly upregulated metabolites in the LNT group.

### 3.7. Metabolite Set Enrichment Analysis and Metabolic Pathway Analysis

**Figure 5:**
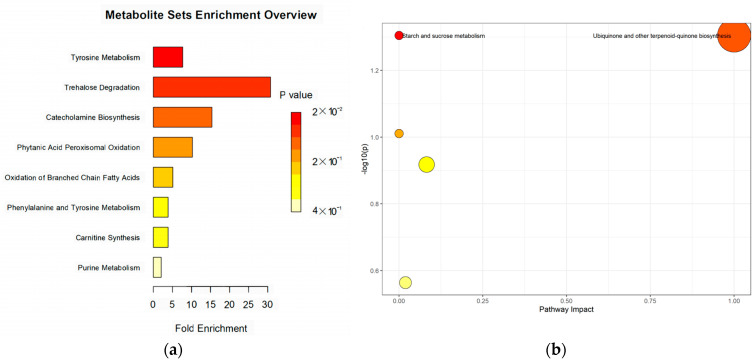
(**a**) Metabolite set enrichment analysis obtained for the metabolite set fold enrichment and *p*-value; and (**b**) analysis of the KEGG pathway enriched by differential metabolites.

As shown in Figure 5a, the enrichment pathways for differential metabolites include tyrosine metabolism and trehalose degradation, with enrichment multiples of 7.69 and 30.77, respectively. Figure 5b shows a metabolic pathway with the significant enrichment of differential metabolites, ubiquinone and other terpene quinone biosynthesis, and starch and sucrose metabolism, that were enriched significantly in differential metabolites, with a *p*-value of <0.05 and a path impact of 1 and 0, respectively.

### 3.8. Correlation Analysis

**Figure 6:**
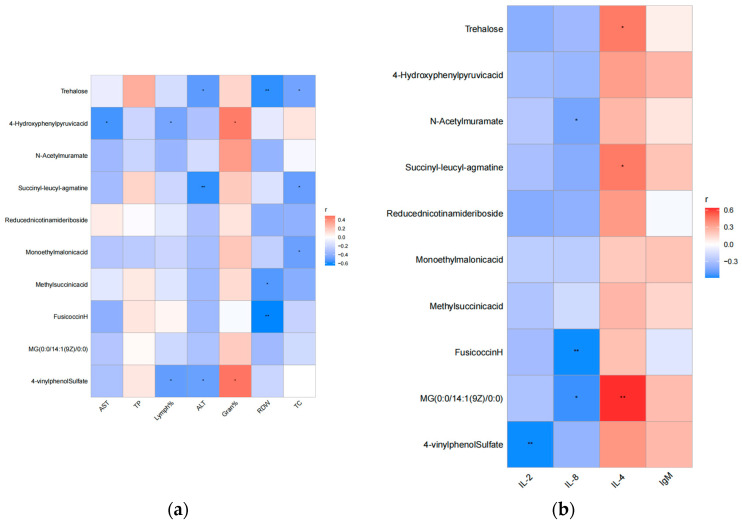
(**a**) Correlation analysis between differential metabolites and hematological parameters, as well as serum biochemical indicators and (**b**) correlation analysis between differential metabolites and immune indicators; * represents 0.01 < *p* ≤ 0.05 and ** represents 0.001 < *p* ≤ 0.01.

The correlation analysis between the differential hematological parameters, blood biochemical indicators, and differential metabolites is shown in Figure 6a. Trehalose was negatively associated with ALT, RDW, and TC (*p* = 0.020, r = −0.514; *p* = 0.008, r = −0.575; *p* = 0.037, r = −0.469). The 4-hydroxyphenylpyruvic acid was negatively associated with AST and Lymph% (*p* = 0.012, r = −0.550; *p* = 0.038, r = −0.468). However, it was positively associated with Gran% (*p* = 0.043, r = 0.456). Succinyl-leucyl-agmatine was negatively associated with ALT and TC (*p* = 0.009, r = −0.571; *p* = 0.026, r = −0.497). Monoethyl malonic acid was negatively associated with TC (*p* = 0.030, r = −0.486). Methylsuccinic acid and fusicoccin H were negatively associated with RDW (*p* = 0.017, r = −0.527; *p* = 0.002, r = −0.639). The 4-vinylphenol sulfate was negatively associated with Lymph% and ALT (*p* = 0.025, r = −0.500; *p* = 0.029, r = −0.488). However, it was positively associated with Gran% (*p* = 0.032, r = 0.480).

The correlation analysis between the differential cytokines and differential metabolites is shown in Figure 6b. Trehalose and succinyl-leucyl-agmatine were positively associated with IL-4 (*p* = 0.047, r = 0.449; *p* = 0.047, r = 0.450). N-acetylmuramate and fusicoccin H were negatively associated with IL-8 (*p* = 0.045, r = −0.454; *p* = 0.008, r = −0.577). MG (0:0/14:1(9Z)/0:0) was negatively associated with IL-8 (*p* = 0.012, r = −0.549). However, it was positively associated with IL-4 (*p* = 0.003, r = 0.623). Lastly, 4-vinylphenol sulfate was negatively associated with IL-2 (*p* = 0.008, r = −0.576).

## 4. Discussion

Dairy cows with higher yields are more prone to mastitis, and hematological parameters could reflect the relevant changes caused by inflammation [16]. In the present research, the addition of 10 g/d of LNT tended to cause a decrease in the number of lymphocytes and led to an increase in the proportion of lymphocytes and a decrease in the proportion of granulocytes. Lymphocytes increase in number during the chronic antigen stimulation involved in infectious diseases, tumors, and adrenal hypofunction. In addition, chronic suppurative diseases such as mastitis can also cause lymphocyte levels to increase [17,18]. In our experiment, the possible reason for the decrease in the number of lymphocytes is that some hormone levels in the serum have changed. Previous research has proven some immune hormones, such as glucocorticoids, which were found to lead to a decrease in the number of lymphocytes [19,20]. Moreover, RDW decreased with the addition of lentinan, which reflects the degree of variation in erythrocyte size. In general, RDW is viewed as a biomarker of some potential conditions, such as inflammation, oxidative damage, impaired liver function, and malnutrition. Roland et al. found that anemia caused by iron and folic acid deficiency could lead to an increase in the index of RDW [18,21]. Thus, we speculate that feeding cows 10 g/d of LNT could improve their hematological parameters and reduce the risk of hematological diseases.

The addition of LNT led to a decrease in the concentration of ALT and AST. ALT is found in various organs and can catalyze the transfer of the α-amino group from alanine to α-ketoglutaric acid. ALT mainly exists in the liver, and it is also considered as one of the indicators used for the detection of liver abnormalities. With the occurrence of liver cell damage, ALT can permeate into the serum, leading to an increase in ALT levels [22,23]. AST is a mitochondrial enzyme involved in the transfer of 2-amino to 2-oxoacid [24]. It exists in multiple tissues and organs, including the liver. Apoptosis, or direct tissue damage, such as plasma membrane damage, is caused by various harmful substances and stimuli. Protein leakage and cell necrosis are the most common factors that increase circulating aminotransferase activity [25]. In addition, in our study, we also found that a low dose of LNT increased the TP content in the cows’ serum. However, we found that the TP content decreased at higher doses. We speculate that the reason for this change was that different doses of β-glucans caused different immune stimulation activities [26]. A high cholesterol content in the serum was one of the factors involved, where it was found to cause cardiovascular diseases in mice [27]. Similarly, high levels of cholesterol were observed in the hypothyroid cows [28]. In the present study, the decline in TC concentration was the result of the action of β-glucans in LNT. It has been reported that various β-glucans have the ability to inhibit cholesterol, with them being able to interact with lipids and bile salts in the intestine, thereby reducing cholesterol levels [29,30,31].

High-yield dairy cows are more likely to suffer from subclinical mastitis, and the level of cytokines in their serum usually reflects the degree of inflammation suffered. It has been reported that cows with subclinical mastitis have higher serum IL-6 levels and lower IL-4 and IL-10 levels [32]. In the current study, the changes in IL-4, IL-6, and IL-10 were consistent with previous experiments, which proves that lentinan is beneficial for reducing subacute mastitis. In addition, IL-1β, IL-2, IL-8, and TNF-α all declined to varying degrees following the addition of LNT. IL-1β, IL-2, IL-8, and TNF-α have been categorized as typical type 2 helper T-lymphocyte (Th1) cytokines, while IL-4 and IL-10 are typical type 2 helper T-lymphocyte (Th2) cytokines. The decrease in the number of Th1 cells and the increase in the number of Th2 cells indicated that LNT promoted the development of Th2 cells, thereby inhibiting the activity of Th1 cells [33]. In a previous study, researchers reported that β-glucans upregulated the anti-inflammatory cytokine IL-10 and inhibited T-cell proliferation and functional differentiation, which might further inhibit the expression of pro-inflammatory factors such as IL-1β, IL-2, IL-8, and TNF-α [34]. Moreover, the addition of LNT adjusted the balance between M1 macrophages and M2 macrophages. In a related study, M2 macrophages inhibited the expression of M1 macrophages, with them producing proinflammatory cytokines by producing anti-inflammatory cytokines such as IL-4 and IL-10 [35]. In another study, Wang et al. found that LNT inhibited proinflammatory cytokine (TNF-α, IL-1β, and IL-6) expression in piglets [36]. Consistent with the results of this study, Nishitani et al. found that lentinan can inhibit the expression of IL-2 and IL-8 [37]. However, in other studies on LNT, LNT stimulated the expression of Th1 to improve the expression of proinflammatory factors [38,39]. We speculate that these differences were caused by varying degrees of inflammation. In the present study, LNT induced a dose-dependent increase in IgM, which is related to the ability of LNT to induce humoral immunity [40]. However, there was no difference in the level of SOD, GSH-Px, and MDA, which may be caused by the following reason. Oxidative stress in cows could weaken gradually after the transition period, and we collected the blood sample at the 117th day of lactation. Previous research has proven that some serum oxidative stress biomarkers, including SOD, GSH-Px, and MDA, would decrease with increasing lactation days [41,42].

Supplementation with LNT further induced changes in metabolic activity, and these differential metabolites were mainly enrichment in the tyrosine metabolism and trehalose degradation. Increased levels of 4-hydroxyphenylpyruvic acid in the blood leads to the upregulation of tyrosine metabolism, which is mainly carried out in the liver. The first step involved in this process is the conversion of cytosolic tyrosine aminotransferase to 4-hydroxyphenylpyruvic acid [43]. Levine et al. and Tessari et al. reported that the concentration of tyrosine in plasma increased when the liver was damaged [44,45]. These findings explain the reasoning as to why plasma AST was negatively correlated with 4-hydroxyphenylpyruvic acid in the current study, indicating that LNT supplementation protected the cows’ livers by upregulating tyrosine metabolism. The significant upregulation of trehalose degradation following the addition of LNT was due to the increase in trehalose. Trehalose is considered one of the main energy sources of most organisms, with it maintaining the structural integrity of the cytoplasm under extreme environmental conditions [46,47]. It has been reported that trehalose can reduce the markers of oxidative stress in dairy cows [48]; however, this finding was not reflected in the results of the current study. We suspect that the dairy cows were not in a state of oxidative stress at the stage in question. In addition, trehalose has been reported to have immune-stimulatory effects, which could cause the activation of macrophages and the release of cytokines, such as IL-4 [49]. In a study, it was able to enhance insulin-stimulated Akt phosphorylation in the liver to protect the organ and reduce serum AST and ALT levels [50]. This might, therefore, be the reason for the negative correlation between trehalose and ALT in the current study. Consistent with the results of this study, Li et al. state that increased levels of trehalose can reduce TC levels because trehalose is able to inhibit cholesterol synthesis genes such as hmgcs and srebp2, and this may also be related to increased trehalose levels leading to reduced glucose uptake and catabolism [51]. Overall, supplementation with LNT can also protect the cow’s liver by upregulating trehalose degradation. Regarding metabolic pathways, supplementation with LNT upregulated the two metabolic pathways of ubiquinone and other terpene quinone biosynthesis, as well as starch and sucrose metabolism. Although the difference between starch and sucrose metabolism was significant, the pathway impact was zero, indicating that the changes were not important in the entire metabolic pathway network. The pathway impact of ubiquinone and other terpene quinone biosynthesis was one, indicating that the changes had a significant impact on the entire metabolic pathway network. The upregulation of 4-hydroxyphenylpyruvic acid was found to be the main factor affecting the pathway. Ubiquinone and other terpene qui-none biosynthesis were related to antioxidant, anti-inflammatory, and immunomodulatory effects. Compared to pathological conditions, the pathway was upregulated when the subject was of a healthier physiological condition [52,53]. In contrast, in dairy cows, ubiquinone and other terpene quinone biosynthesis were upregulated in high-yield cows in comparison to low-yield dairy cows [54]. In the present study, the upregulation of this pathway implied that LNT improved the physiological condition of the dairy cows.

Among the remaining differential metabolites that do not possess an enrichment set of metabolites, 4-vinylphenol sulfate, fusicoccin H, and methylsuccinic acid are extracts or metabolites of fungi, and their upregulation in the LNT group might be caused by feeding [55,56,57]. The increase in reduced nicotinamide riboside led to the enhancement of tissue physiological functions. In studies carried out in recent years, reduced nicotinamide riboside has been found to be a more efficient and rapid precursor of NAD+ biosynthesis, with it being able to prevent, alleviate, and even reverse various metabolic diseases [58]. The reasons for the upregulation of succinyl-leucyl-agmatin, MG(0:0/14:1(9Z)/0:0), and N-acetylmuramate are still unclear. According to the results of our correlation analysis, they inhibited the production of proinflammatory factors and promoted the production of anti-inflammatory factors. In addition, succinyl-leucyl-agmatin was found to reduce the production of AST and TC, which may have a specific protective effect on the liver.

## 5. Conclusions

Supplementation with 10 g/d of LNT can improve the hematological parameters of dairy cows, reduce the levels of ALT, AST, IL-2, and IL-8 in their serum, and increase the levels of anti-inflammatory factors such as IL-4. Regarding the results of the metabolomic analysis, LNT changed the metabolites in the plasma of the dairy cows, with most of them being enriched in tyrosine metabolism and trehalose degradation. They upregulated ubiquinone and other terpene quinone biosynthesis, and starch and sucrose metabolism in these two pathways, thus improving the immunity of the dairy cows.

## Figures and Tables

**Table 1:** Ingredients and chemical composition of the basal diet.

Ingredient	Concentration (%)	Nutritional Composition	Content (%)
Wheat whole-plant silage	20.80	Dry matter	55.21
Alfalfa hay	6.10	Crude protein	17.19
Chinese wildrye	10.50	Neutral detergent fibers	38.44
High-energy forage ^1^	2.20	Acid detergent fiber	20.97
Distiller grains	4.30	Ether extract	2.46
Potato skin	8.00	Ca	1.06
CaHPO_4_	0.40	P	0.44
Limestone	0.40	NE_L_/(MJ/kg) ^4^	7.06
NaHCO_3_	0.10		
NaCl	0.10		
TMR high-yield concentrate ^2^	45.10		
Premix ^3^	2.00		
Total	100.00		

^1^ Low-fiber straw. ^2^ The mixed concentrate was composed of 40.0% corn, 6.0% wheat, 9.6% soybean meal, 8.0% bran, 2.5% cotton meal, 6.0% rapeseed meal, 2.0% rice bran, 4.2% DDGS, 8.0% rice bran meal, 1.5% biological protein feed, 5.5% sesame meal, etc. ^3^ The 1 kg premix contained the following ingredients: VA 200,000 IU, VD 60,000 IU, Fe 400 mg, Cu 450 mg, Zn 2000 mg, Mn 550 mg, Se 15 mg, and Co 20 mg. ^4^ NE_L_ was calculated according to NRC (2001) [13].

**Table 2:** Effect of LNT supplementation on the hematological parameters of the dairy cows.

Item	Treatment				Reference Values	SEM	*p*-Value
	CON	LL	ML	HL			
WBC, ×10^9^/L	20.31	13.76	11.74	14.40	5.0–16.0	1.343	0.124
Mon, ×10^9^/L	0.99	0.73	0.69	0.73	0.3–1.6	0.065	0.221
Lymph, ×10^9^/L	12.81	8.10	5.31	8.19	1.5–9.0	1.032	0.069
Gran, ×10^9^/L	6.51	4.93	5.74	5.49	2.3–9.1	0.361	0.507
Lymph, %	60.41 ^a^	55.96 ^a^	44.89 ^b^	54.53 ^ab^	20.0–60.3	1.922	0.023
Mon, %	5.17	5.73	6.04	5.84	4.0–12.1	0.217	0.528
Gran, %	34.41 ^a^	38.31 ^a^	49.07 ^b^	39.63 ^a^	30.0–65.0	1.784	0.018
RBC, ×10^12^/L	6.34	6.18	6.39	6.69	5.00–10.10	0.242	0.907
HGB, g/L	102.43	94.29	104.14	103.71	90–139	3.977	0.815
HCT, %	30.51	28.59	30.73	31.29	28.0–46.0	1.184	0.878
MCV, fL	48.63	49.01	48.07	46.87	38.0–53.0	0.642	0.682
MCH, pg	16.17	16.00	16.20	15.66	13.0–19.0	0.206	0.770
MCHC, g/L	334.71	329.14	340.00	336.19	300–370	2.192	0.383
RDW, %	16.40 ^a^	15.51 ^ab^	15.11 ^b^	15.23 ^b^	14.0–19.0	0.178	0.026
PLT, ×10^9^/L	456.86	409.71	482.57	422.57	120–820	28.382	0.815
PDW	15.87	15.71	15.91	15.73	14.0–19.0	0.081	0.774
MPV, fL	5.81	5.77	5.86	5.67	3.8–7.0	0.083	0.882
PCT, %	0.26	0.23	0.28	0.24	0.12–0.42	0.026	0.741

^a,b^ Within a row, means without a common superscript differ at *p* < 0.05.

**Table 3:** Effect of LNT supplementation on the serum biochemical indices of the dairy cows.

Item	Treatment				Reference Values	SEM	*p*-Value
	CON	LL	ML	HL			
ALT, U/L	47.11 ^a^	40.30 ^b^	37.33 ^b^	47.01 ^a^		1.140	<0.001
AST, U/L	111.24 ^a^	98.43 ^ab^	84.09 ^b^	100.70 ^ab^	50.0–150.0	3.242	0.020
ALP, U/L	60.66	61.87	48.97	57.29	28.0–233.0	2.636	0.313
TP, g/L	71.13 ^ab^	74.01 ^a^	68.63 ^b^	71.70 ^ab^	62.0–80.0	0.776	0.029
ALB, g/L	31.70	30.49	30.04	30.93	25.0–35.0	0.265	0.145
GLB, g/L	39.33	43.52	38.59	39.86	30.0–49.0	0.774	0.074
A/G	0.81	0.71	0.79	0.77		0.017	0.496
UREA, mmol/L	5.30	5.05	5.18	5.33	3.60–9.30	0.088	0.688
GLU, mmol/L	3.95	4.13	3.80	4.06	3.11–4.89	0.054	0.144
Ca, mmol/L	2.58	2.52	2.46	2.51	2.00–3.00	0.018	0.161
P, mmol/L	1.77	1.55	1.65	1.51	1.30–2.80	0.051	0.298
TC, mmol/L	7.97 ^a^	7.25 ^ab^	6.21 ^b^	6.79 ^b^	1.20–5.20	0.209	0.014
TG, mmol/L	0.10	0.09	0.08	0.08	≤0.10	0.004	0.111

^a,b^ Within a row, means without a common superscript differ at *p* < 0.05.

**Table 4:** Effect of LNT supplementation on the serum immune indices of dairy cows.

Item	Treatment				SEM	*p*-Value
	CON	LL	ML	HL		
IL-1β, pg/mL	109.47	104.72	58.97	58.20	9.356	0.070
IL-2, pg/mL	108.43 ^a^	90.10 ^ab^	68.86 ^b^	67.20 ^b^	5.824	0.027
IL-4, pg/mL	8.04 ^b^	11.84 ^a^	11.95 ^a^	11.39 ^a^	0.577	0.039
IL-6, pg/mL	96.02	134.68	84.31	82.54	8.511	0.099
IL-8, pg/mL	59.02 ^a^	41.20 ^b^	43.29 ^b^	44.86 ^b^	2.401	0.026
IL-10, pg/mL	30.20	47.86	50.38	39.19	3.124	0.083
IL-11, pg/mL	128.75	147.49	154.96	141.31	6.738	0.590
IgA, μg/mL	38.19	42.63	37.25	48.40	2.442	0.376
IgG, μg/mL	178.66	224.82	172.25	198.05	11.932	0.133
IgM, μg/mL	7.98 ^b^	10.05 ^ab^	10.67 ^ab^	13.77 ^a^	0.613	0.005
TGF-β1, pg/mL	124.07	173.61	157.71	157.96	9.419	0.298
TNF-α, pg/mL	49.56	58.59	36.59	37.36	3.499	0.077

^a,b^ Within a row, means without a common superscript differ at *p* < 0.05.

**Table 5:** Effect of LNT supplementation on the serum immune indices of the dairy cows.

Item	Treatment				SEM	*p*-Value
	CON	LL	ML	HL		
SOD, U/mL	133.13	187.93	148.78	150.61	8.121	0.106
GSH-Px, IU/mL	143.24	135.03	107.04	115.82	11.990	0.701
MDA, nmol/mL	3.06	3.17	2.57	3.37	0.162	0.342

## Data Availability

Due to restrictions, the data are available upon request.

## References

[1] Giavasis I. (2014). Bioactive fungal polysaccharides as potential functional ingredients in food and nutraceuticals. Curr. Opin. Biotech..

[2] Yoshino S., Nishikawa K., Morita S., Takahashi T., Sakata K., Nagao J., Nemoto H., Murakami N., Matsuda T., Hasegawa H. (2016). Randomised phase III study of S-1 alone versus S-1 plus lentinan for unresectable or recurrent gastric cancer (JFMC36-0701). Eur. J. Cancer..

[3] Han X., Luo R., Ye N., Hu Y., Fu C., Gao R., Fu S., Gao F. (2022). Research progress on natural β-glucan in intestinal diseases. Int. J. Biol. Macromol..

[4] Zhang Y., Zhang M., Jiang Y., Li X., He Y., Zeng P., Guo Z., Chang Y., Luo H., Liu Y. (2018). Lentinan as an immunotherapeutic for treating lung cancer: A review of 12 years clinical studies in China. J. Cancer. Res Clin..

[5] Murphy E.J., Rezoagli E., Pogue R., Simonassi-Paiva B., Abidin I.I.Z., Fehrenbach G.W., O’Neil E., Major I., Laffey J.G., Rowan N. (2022). Immunomodulatory activity of β-glucan polysaccharides isolated from different species of mushroom–A potential treatment for inflammatory lung conditions. Sci. Total. Environ..

[6] Jo W.S., Choi Y.J., Mm H.J., Lee J.Y., Nam B.H., Lee J.D., Lee S.W., Seo S.Y., Jeong M.H. (2010). The anti-inflammatory effects of water extract from Cordyceps militaris in murine macrophage. Mycobiology.

[7] Du B., Lin C., Bian Z., Xu B. (2015). An insight into anti-inflammatory effects of fungal beta-glucans. Trends. Food. Sci. Technol..

[8] Ahn H., Jeon E., Kim J.C., Kang S.G., Yoon S.I., Ko H.J., Kim P.H., Lee G.S. (2017). Lentinan from shiitake selectively attenuates AIM2 and non-canonical inflammasome activation while inducing pro-inflammatory cytokine production. Sci. Rep..

[9] Ren G., Yu M., Li K., Hu Y., Wang Y., Xu X., Qu J. (2016). Seleno-lentinan prevents chronic pancreatitis development and modulates gut microbiota in mice. J. Funct. Foods..

[10] Ren G., Xu L., Lu T., Zhang Y., Wang Y., Yin J. (2019). Protective effects of lentinan on lipopolysaccharide induced inflammatory response in intestine of juvenile taimen (Hucho taimen, Pallas). Int. J. Biol. Macromol..

[11] Ma T., Tu Y., Zhang N., Deng K., Zhou Z., Yun Q., Diao Q. (2015). Effects of dietary yeast β-glucan on nutrient digestibility and serum profiles in pre-ruminant Holstein calves. J. Integr. Agr..

[12] Poławska E., Bagnicka A.W., Niemczuk K., Lipińska J.O. (2012). Relations between the oxidative status, mastitis, milk quality and disorders of reproductive functions in dairy cows—A review. Anim. Sci. Pap. Rep..

[13] NRC (National Research Council) (2001). Nutrient Requirements of Dairy Cattle.

[14] Aiello S.E., Moses M.A. (2016). The Merck Veterinary Manual.

[15] Wang J. (1999). Veterinary Internal Medicine.

[16] Seegers H., Fourichon C., Beaudeau F. (2003). Production effects related to mastitis and mastitis economics in dairy cattle herds. Vet. Res..

[17] Leitner G., Shoshani E., Krifucks O., Chaffer M., Saran A. (2000). Milk leucocyte population patterns in bovine udder infection of different aetiology. J. Vet. Med. B..

[18] Roland L., Drillich M., Iwersen M. (2014). Hematology as a diagnostic tool in bovine medicine. J. Vet. Diagn. Investig..

[19] Meglia G.E., Johannisson A., Agenäs S., Holtenius K., Waller K.P. (2005). Effects of feeding intensity during the dry period on leukocyte and lymphocyte sub-populations, neutrophil function and health in periparturient dairy cows. Vet. J..

[20] Spies C.M., Strehl C., van der Goes M.C., Bijlsma J.W., Buttgereit F. (2011). Glucocorticoids. Best. Pract. Res. Cl. Rh..

[21] Lippi G., Plebani M. (2014). Red blood cell distribution width (RDW) and human pathology. One size fits all. Clin. Chem. Lab. Med..

[22] Sherman K.E. (1991). Alanine aminotransferase in clinical practice: A review. Arch. Intern. Med..

[23] Kaplan M.M. (2002). Alanine aminotransferase levels: What’s normal?. Ann. Intern. Med..

[24] Whitehead M.W., Hawkes N.D., Hainsworth I., Kingham J.G.C. (1999). A prospective study of the causes of notably raised aspartate aminotransferase of liver origin. Gut.

[25] Ndrepepa G. (2021). Aspartate aminotransferase and cardiovascular disease—A narrative review. J. Lab. Precis. Med..

[26] Bagni M., Romano N., Finoia M.G., Abelli L., Scapigliati G., Tiscar P.G., Sarti M., Marino G. (2005). Short-and long-term effects of a dietary yeast β-glucan (Macrogard) and alginic acid (Ergosan) preparation on immune response in sea bass (*Dicentrarchus labrax*). Fish. Shellfish. Immun..

[27] Damodharan K., Palaniyandi S.A., Yang S.H., Suh J.W. (2016). Functional probiotic characterization and in vivo cholesterol-lowering activity of Lactobacillus helveticus isolated from fermented cow milk. J. Microbiol. Biotechn..

[28] Lennon J.H.D., Mixner J.P. (1957). Some sources of variation in total plasma cholesterol levels in dairy cattle. J. Dairy. Sci..

[29] Bell S., Goldman V.M., Bistrian B.R., Arnold A.H., Ostroff G., Forse R.A. (1999). Effect of β-glucan from oats and yeast on serum lipids. Crit. Rev. Food. Sci..

[30] Othman R.A., Moghadasian M.H., Jones P.J. (2011). Cholesterol-lowering effects of oat β-glucan. Nutr. Rev..

[31] Sima P., Vannucci L., Vetvicka V. (2018). β-glucans and cholesterol. Int. J. Mol. Med..

[32] Bochniarz M., Zdzisińska B., Wawron W., Szczubiał M., Dąbrowski R. (2017). Milk and serum IL-4, IL-6, IL-10, and amyloid A concentrations in cows with subclinical mastitis caused by coagulase-negative staphylococci. J. Dairy. Sci..

[33] Volman J.J., Ramakers J.D., Plat J. (2008). Dietary modulation of immune function by β-glucans. Physiol. Behav..

[34] Vetvicka V., Vannucci L., Sima P. (2014). The effects of β-glucan on pig growth and immunity. Open. Biochem. J..

[35] Liao H., Ye J., Gao L., Liu Y. (2021). The main bioactive compounds of Scutellaria baicalensis Georgi. for alleviation of inflammatory cytokines: A comprehensive review. Biomed. Pharmacother..

[36] Wang X., Wang W., Wang L., Yu C., Zhang G., Zhu H., Wang C., Zhao S., Hu C.A., Liu Y. (2019). Lentinan modulates intestinal microbiota and enhances barrier integrity in a piglet model challenged with lipopolysaccharide. Food. Funct..

[37] Nishitani Y., Zhang L., Yoshida M., Azuma T., Kanazawa K., Hashimoto T., Mizuno M. (2013). Intestinal anti-inflammatory activity of lentinan: Influence on IL-8 and TNFR1 expression in intestinal epithelial cells. PLoS ONE.

[38] Zhang Q.H., Zhang Y., Liu J., Cao Y.M. (2009). The shiitake mushroom-derived immuno-stimulant lentinan protects against murine malaria blood-stage infection by evoking adaptive immune-responses. Int. Immunopharmacol..

[39] Wang X.E., Wang Y.H., Zhou Q., Peng M., Zhang J., Chen M., Ma L.J., Xie G.M. (2020). Immunomodulatory effect of lentinan on aberrant T subsets and cytokines profile in non-small cell lung cancer patients. Pathol. Oncol. Res..

[40] Zhang Q., Cong R., Hu M., Zhu Y., Yang X. (2017). Immunoenhancement of edible fungal polysaccharides (lentinan, tremellan, and pachymaran) on cyclophosphamide-induced immunosuppression in mouse model. Evid-Based. Compl. Alt..

[41] Sharma N., Singh N.K., Singh O.P., Pandey V., Verma P.K. (2011). Oxidative stress and antioxidant status during transition period in dairy cows. Asian. Austral. J. Anim..

[42] Mirzad A.N., Tada T., Ano H., Kobayashi I., Yamauchi T., Katamoto H. (2018). Seasonal changes in serum oxidative stress biomarkers in dairy and beef cows in a daytime grazing system. J. Vet. Med. Sci..

[43] Chakrapani A., Gissen P., McKiernan P., Saudubray J.M., Baumgartner M.R., García-Cazorla Á., Walter J. (2016). Disorders of tyrosine metabolism. Inborn Metabolic Diseases: Diagnosis and Treatment.

[44] Levine R.J., Conn H.O. (1967). Tyrosine metabolism in patients with liver disease. J. Clin. Investig..

[45] Tessari P., Vettore M., Millioni R., Puricelli L., Orlando R. (2010). Effect of liver cirrhosis on phenylalanine and tyrosine metabolism. Curr. Opin. Clin. Nutr..

[46] Wiemken A. (1990). Trehalose in yeast, stress protectant rather than reserve carbohydrate. Anton. Leeuw..

[47] Higashiyama T. (2003). Novel functions and applications of trehalose. Pure. Appl. Chem..

[48] Aoki N., Furukawa S., Sato K., Kurokawa Y., Kanda S., Takahashi Y., Mitsuzumi H., Itabashi H. (2010). Supplementation of the diet of dairy cows with trehalose results in milk with low lipid peroxide and high antioxidant content. J. Dairy. Sci..

[49] Vanaporn M., Titball R.W. (2020). Trehalose and bacterial virulence. Virulence.

[50] Stachowicz A., Wiśniewska A., Kuś K., Kiepura A., Gębska A., Gajda M., Białas M., Totoń-Żurańska J., Stachyra K., Suski M. (2019). The influence of trehalose on atherosclerosis and hepatic steatosis in apolipoprotein E knockout mice. Int. J. Mol. Sci..

[51] Li R.X., Chen L.Y., Yao B., Rahimnejad S., Ren J., Luo Y., Qiao F., Zhang M.L., Du Z.Y. (2022). Trehalose alleviated hepatic cholesterol accumulation via inhibiting transformation from glucose-derived acyl-CoA to cholesterol synthesis in Nile tilapia. Aquaculture.

[52] Lee K.H., Guo J., Song Y., Ariff A., O’Sullivan M., Hales B., Mullins B.J., Zhang G. (2021). Dysfunctional gut microbiome networks in childhood IgE-Mediated food allergy. Int. J. Mol. Sci..

[53] Zhen W., Liu Y., Shao Y., Ma Y., Wu Y., Guo F., Abbas W., Guo Y., Wang Z. (2021). Yeast β-glucan altered intestinal microbiome and metabolome in older hens. Front. Microbiol..

[54] Mu Y., Lin X., Wang Z., Hou Q., Wang Y., Hu Z. (2019). High-production dairy cattle exhibit different rumen and fecal bacterial community and rumen metabolite profile than low-production cattle. MicrobiologyOpen.

[55] Chesters N.C., O’Hagan D. (1997). Biosynthesis of the fungal metabolite, piliformic acid (2-hexylidene-3-methylsuccinic acid). J. Chem. Soc. Perkin Trans. 1.

[56] Kamiyama M., Horiuchi M., Umano K., Kondo K., Otsuka Y., Shibamoto T. (2013). Antioxidant/anti-inflammatory activities and chemical composition of extracts from the mushroom Trametes versicolor. Int. J. Nutr. Food. Sci..

[57] Evidente A., Kornienko A., Cimmino A., Andolfi A., Lefranc F., Mathieu V., Kiss R. (2014). Fungal metabolites with anticancer activity. Nat. Prod. Rep..

[58] Giroud-Gerbetant J., Joffraud M., Giner M.P., Cercillieux A., Bartova S., Makarov M.V., Zapata-Pérez R., Sánchez-García J.L., Houtkooper R.H., Migaud M.E. (2019). A reduced form of nicotinamide riboside defines a new path for NAD+ biosynthesis and acts as an orally bioavailable NAD+ precursor. Mol. Metab..

